# Supplementary material for: The Role of Pigments and Cryptochrome 1 in the Adaptation of Solanum lycopersicum Photosynthetic Apparatus to High-Intensity Blue Light
Source: Antioxidants (Basel). 2024 May 15;13(5):605. doi: 10.3390/antiox13050605 (PMC11117525; doi:10.3390/antiox13050605)
Supplement: Supplementary file 1 [file antioxidants-13-00605-s001.zip › antioxidants-2996804-supplementary/Table S1.pdf]

Table S1. Primers for qRT-PCR analysis

	NCBI RefSeq	Gene	Gene description	Forward	Reverse
1	NM_00124747191.2	*HY5*	Transcription factor HY5	AAGAGAGGAAGAAGCCCAGC	CTCTCCCTTGCTTGTTGTGC
2	AH009615.2	*POR1*	Protochlorophyllide oxidoreductase (SlPOR)	CTTCATTCAAAATCAAGAGCGGAAG	TTCTTTCCTGTCACAGGGGTACTC
3	NM_001320256.1	*ELIP*	Early light inducible protein	CCTTCATAGGACTTCGCGTGTTA	CTTGGTTTTGGCTTAGCAGGT
4	X60275	*CAB1*	LhbC1 gene for LHCII type III	TCAGTGACGGTGGACTTGAC	ACCGGGGTATAAGTCGTTGC
5	NM_001247883.2	*PSY1*	Phytoene synthase 1	CGGGGAATTTGGGCTTGTTG	CCACCTATCTAAGGCTGCCG
6	XM_004249510.4	*PAL1*	Phenylalanine ammonia-lyase 1	GAAGCGTTCCATGTTGCTGG	TCAGTGAACTCGGGCTTTCC
7	NM_001247104.2	*CHS*	Chalcone synthase	CCGTGGACCCAGTGAATCTC	AGAGTTTGGGCTGCTGAGAC
8	NM_001374394.1	*ANS*	Anthocyanidin synthase	TCTCAATTCCCACCTCGCAC	ACTTTGCGCTCAGCAAGAAC
9	L14403.1	*rbcL*	Ribulose-1,5-bisphosphate carboxylase/oxygenase large subunit	CTTTCCAAGGTCCGCCTCAT	AAGTCCACCGCGAAGACATT
11	AM087200.3	*psbA*	Photosystem II protein D1	AATAGGGAGCCGCCGAATAC	AGCTGCTACCGCAGTTTTCT
12	AM087200.3	*psbD*	Photosystem II protein D2	TATGATGGGAGTTGCCGGTG	TCTTCGGCTTGAGTTGGGTT
13	AM087200.3	*psbB*	Photosystem II CP47 chlorophyll-binding protein	TCTTGGCAAAGGCCTCAAGT	CTGCCTAGCGGAACGGTTA
14	AM087200.3	*psbC*	Photosystem II CP43 chlorophyll-binding protein	GCACTTCTGGGACCTGAGAC	TCTTACATCTCCCCCTCCCG
15	Z11999.1	*psbO*	Photosystem II manganese-stabilizing protein	AACAAGGTTCAGGCAGGCTT	ACTCCTTCCGCATTTGCTCC
16	X63007.1	*psbP*	oxygen evolving enhancer 2 of Photosystem II	TACGGCTCCCCTGAAGAGTT	GCCTCCAACAGGTTAGCAGT
17	AY568719.1	*psbQ*	Photosystem II oxygen-evolving complex protein 3	GGCAGCCCAGAGAGTTAAGG	GCTGCATGGTCCAGATCACT
18	A0A3Q7F8W6	*Tubulin1*	Tubulin alpha chain	ACAACTTTGCCCGTGGACAT	TGCTCAAGAAGGGAGTGGGT
