# Supplementary figures and images for: The Role of Pigments and Cryptochrome 1 in the Adaptation of Solanum lycopersicum Photosynthetic Apparatus to High-Intensity Blue Light
Source: Antioxidants (Basel). 2024 May 15;13(5):605. doi: 10.3390/antiox13050605 (PMC11117525; doi:10.3390/antiox13050605)

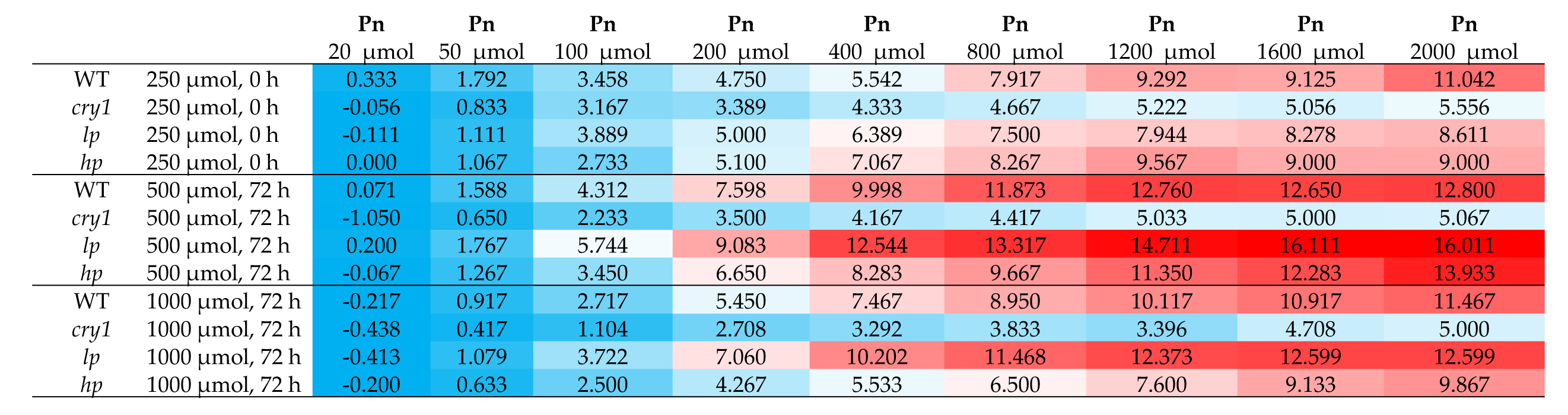

Supplement: Supplementary file 1 [file antioxidants-13-00605-s001.zip › antioxidants-2996804-supplementary/Figure S1A.jpg]

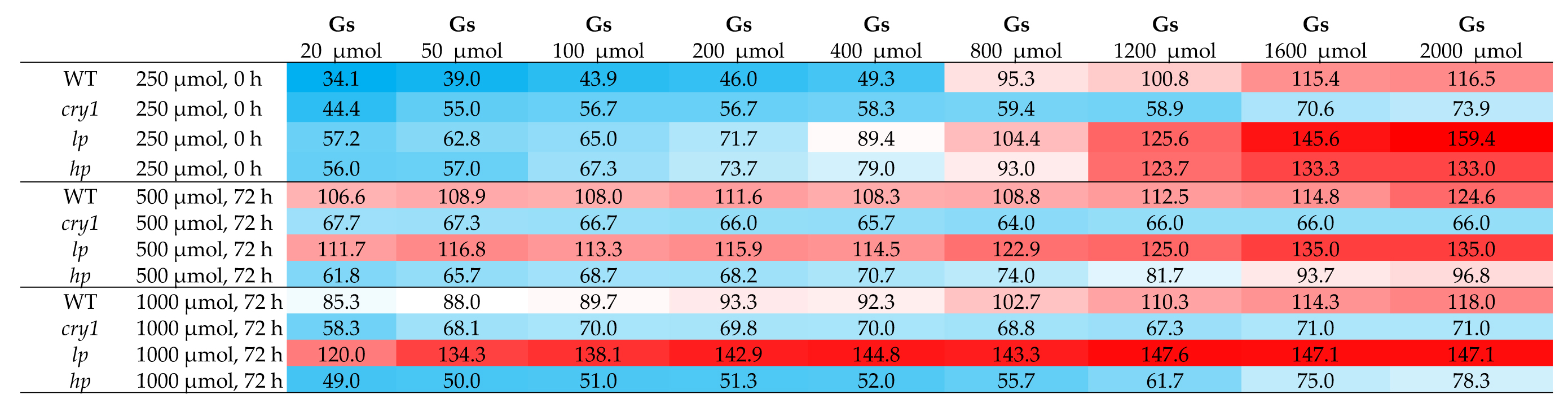

Supplement: Supplementary file 1 [file antioxidants-13-00605-s001.zip › antioxidants-2996804-supplementary/Figure S1B.jpg]

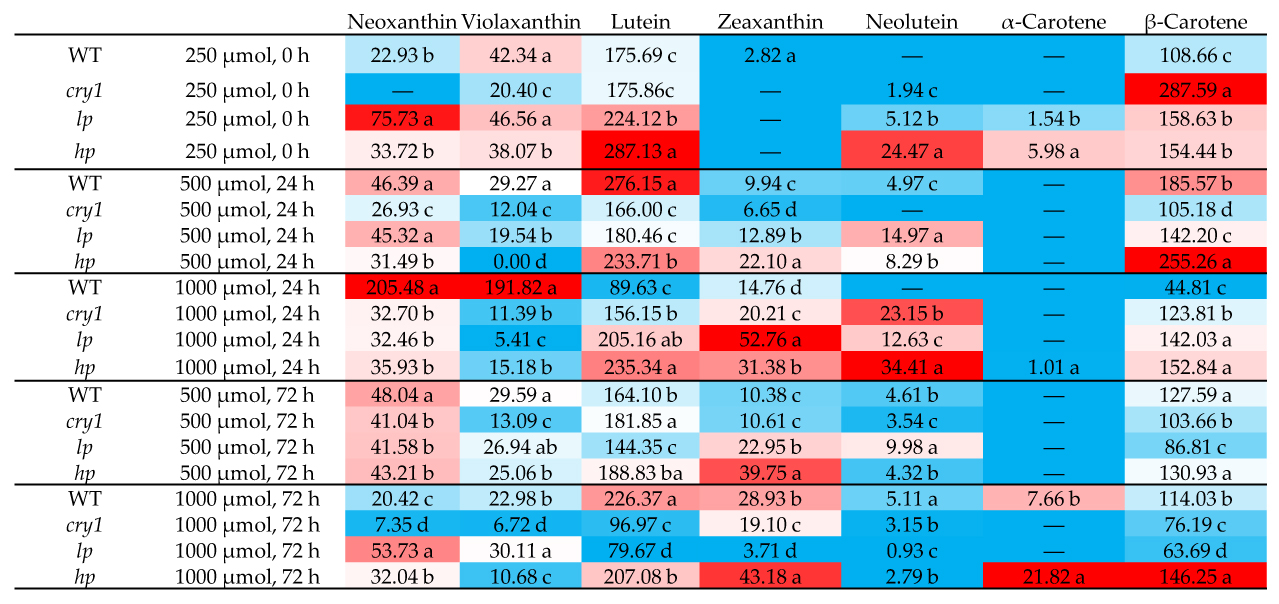

Supplement: Supplementary file 1 [file antioxidants-13-00605-s001.zip › antioxidants-2996804-supplementary/Figure S2.jpg]
